# Supplementary material for: Effectiveness and Safety of Ayurvedic Medicines in Type 2 Diabetes Mellitus Management: A Systematic Review and Meta-Analysis
Source: Front Pharmacol. 2022 Jun 8;13:821810. doi: 10.3389/fphar.2022.821810 (PMC9213670; doi:10.3389/fphar.2022.821810)
Supplement: Supplementary file 1 [file DataSheet1.zip › Supplementary Material/Forest and Funnel Plots/Punica granatum/Fasting insulin.pdf]

Punica granatum - Fasting insulin

Study	Intervention N	Intervention Mean	Intervention SD	Comparator N	Comparator Mean	Comparator SD	Mean Diff. with 95% CI	Weight (%)
Babaeian 2013	23	9.58	5.11	22	11.65	6.44	-2.07 [-5.46, 1.32]	12.37
Sohrab 2014, Sohrab 2015	22	7.10	2.60	22	7.10	3.10	0.00 [-1.69, 1.69]	49.71
Faghihimani 2017	37	9.80	5.40	37	10.10	5.50	-0.30 [-2.78, 2.18]	23.04
Khajebishak 2019a, Khajebishak 2019b	26	13.32	7.87	26	11.72	10.11	1.60 [-3.32, 6.52]	5.86
Grabež 2020	19	11.74	6.44	18	13.72	5.84	-1.98 [-5.95, 1.99]	9.02
**Overall**							-0.41 [-1.60, 0.78]	

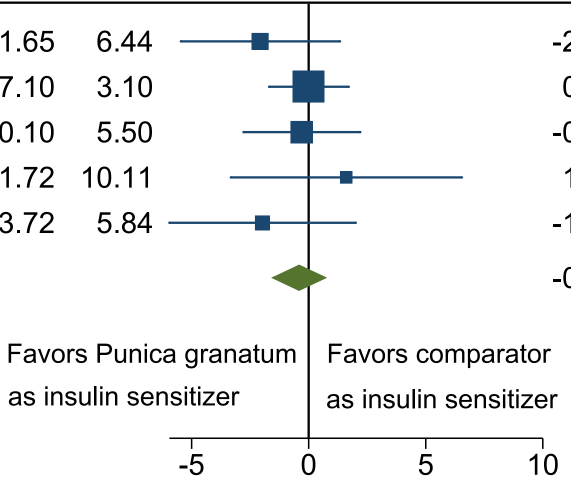

Heterogeneity: $\tau^2 = 0.00$, $I^2 = 0.00\%$, $H^2 = 1.00$

Test of $\theta_i = \theta_j$: Q(4) = 2.40, p = 0.66

Test of $\theta = 0$: z = -0.67, p = 0.50

Random-effects REML model
